# Identification of Low- versus High-Risk Acute Coronary Syndrome for a Selective ECG Monitoring Strategy

**DOI:** 10.3390/jcm12144604

**Published:** 2023-07-11

**Authors:** Mariama Akodad, Pierre-Alain Meunier, Caroline Padovani, Guillaume Cayla, Wassim Zitouni, Jean-Christophe Macia, Pierre Robert, Matthieu Steinecker, François Roubille, Florence Leclercq

**Affiliations:** 1 South Paris Cardiovascular Institute, Jacques Cartie Hospital, 91300 Massy, France; akodadmyriam@gmail.com; 2 Department of Cardiology, University Hospital of Montpellier, 34295 Montpellier, France; pierrealain.meunier@gmail.com (P.-A.M.); c-padovani@chu-montpellier.fr (C.P.); w-zitouni@chu-montpellier.fr (W.Z.); jc-macia@chu-montpellier.fr (J.-C.M.); matthieu-steinecker@chu-montpellier.fr (M.S.); francois.roubille@gmail.com (F.R.); 3 Department of Cardiology, University Hospital of Nîmes, 30900 Nîmes, France; cayla.guillaume@gmail.com (G.C.); pierrecardio@gmail.com (P.R.)

**Keywords:** risk stratification, acute coronary syndrome, NSTEMI, STEMI, ECG monitoring, cardiology intensive care unit

## Abstract

Background: While admission of patients with acute coronary syndromes (ACS) in cardiology intensive care unit (CICU) is usual, in-hospital major outcomes in lower risk patients may be evaluated after early coronary angiography according to the European guidelines. Methods: Consecutive ACS patients were prospectively included after coronary angiography evaluation within 24 h and percutaneous coronary intervention (PCI), when required. Patients were classified as high- or low-risk according to hemodynamics, rhythmic state, ischemic and bleeding risks. Major in-hospital outcomes were assessed. Results: From January to June 2021, 277 patients were enrolled (62.8% with ST-segment elevation myocardial infarction (STEMI) (n = 174); 37.2% with non-NSTEMI (NSTEMI) (n = 103). PCI was required for 260 patients (93.9%). Seventy-four patients (26.7%) were classified as low-risk (n = 47 NSTEMI; n= 27 STEMI) and 203 patients (73.3%) as high-risk of events. All patients were monitored in CICU. While 38 patients (18.7%) from the high-risk group reached the primary endpoint, mainly related to rhythmic or conduction disorder (n = 24, 11.8%) or unstable hemodynamics (n = 17; 8.4%), only 1 patient (1.3%) in the low-risk group had one major outcome (no fatal bleeding); *p* < 0.01. The negative predictive value of our patient stratification for the absence of major in-hospital outcome was 100% (CI95%: 100–100%) for STEMI and 97.9% [CI95%: 93.2–100%] for NSTEMI patients. Conclusions: Stratification of ACS patients after early coronary angiography and most of the time PCI, identify a population with very low risk of in-hospital events (1/4 of all ACS and 1/2 of NSTEMI) who may probably not require ECG monitoring and/or CICU admission. (NCT04378504).

## 1. Introduction

Acute coronary syndrome (ACS) remains one of the leading causes of death worldwide but the incidence of serious in-hospital adverse events significantly decreased over the past decades mainly due to early percutaneous coronary intervention (PCI) using new generation drugs eluting stents surrounded by optimal antithrombotic therapy [1,2]. Current 2017 European guidelines recommend at least 24 h monitoring in cardiology intensive care unit (CICU) for STEMI patients [3]. Rhythm monitoring up to 24 h or until PCI is being performed (whichever comes first) is recommended in the 2020 European guidelines for NSTEMI management for patients at low-risk for cardiac arrhythmias (grade 1 and level of evidence C) [4]. However, CICU admission in observational studies and registries appears minimally correlated with severity of illness but rather related to CICU availability, with many low-risk patients admitted in CICU while some high-risk patients are not admitted [5,6,7]. While international guidelines have indicated that use of CICU must be favored for high-risk patients with NSTEMI and for all STEMI patients, no prospective study supports this strategy [5,6,7,8,9,10]. With improvements in ACS treatment, routine triage of many patients to the CICU is questionable. Furthermore, optimal utilization of resource intensive setting is becoming a major issue [5,6]. Thus, lower risk ACS patients who benefit from successful early PCI and without significant comorbidity may not require electrocardiogram (ECG) monitoring and/or CICU admission. Evaluation of in-hospital serious adverse events occurring in ACS population with contemporary medical and interventional approach according to guidelines may help to identify patients who may safely benefit from such approach.

The aim of this study was to compare the incidence of serious in-hospital medical events in two groups of ACS patients admitted in CICU and classified as high- or low-risk following early coronary angiography evaluation and PCI when required.

## 2. Materials and Methods

### 2.1. Study Design and Patients

We prospectively included all patients admitted for ACS in our CICU department (Montpellier University Hospital, France) from January to June 2021. NSTEMI was defined according to guidelines as an episode of chest pain with or without ECG modifications and significant troponin elevation (hypersensitive troponin T (hs-cTnT) (Elecsys Roche) ≥52 ng/L or significant variation >10 ng/L between 2 dosages at 3 h interval). All NSTEMI patients had coronary angiography evaluation and PCI when required within the first 24 h following admission according to the 2020 European guidelines [4]. Primary PCI was the preferred reperfusion strategy in all STEMI patients [3]. Exclusion criteria included the absence of coronary angiography regardless of the reason (severe renal failure, patient refusal and end of life patient), unstable angina (absence of troponin elevation), coronary angiography or PCI > 24 h following admission, patients with severe mental disease, patients not residing in France and patients transferred to another hospital before the end of hospitalization.

### 2.2. High vs. Low-Risk Patients

After clinical and angiographic evaluation, patients were then classified as being at high- or low-risk of events according to simple medical criteria derived from guidelines (Table 1). All STEMI patients were included in the high-risk group, except for those who had successful (TIMI 3 flow) and non-complicated coronary reperfusion less than 3 h after the onset of chest pain or with open artery at the initial coronary angiography performed within the first 3 h after the onset of chest pain and with a single vessel disease. For NSTEMI patients, low risk criteria included <80 years old, stable hemodynamics, no severe comorbidities requiring special care, LVEF > 40%, successful PCI when required, complete revascularization when required, low or moderate bleeding risk, and stable rhythmic state (Table 1). All patients were systematically admitted in CICU for monitoring. The CICU in our center is a 15-bed unit with full-time staff consisting of 6 certified doctors and 1 nurse for 4 patients. Our CICU allows continuous ECG monitoring, hemodynamics, and clinical monitoring as well as non-invasive ventilation support. An ECG was performed at least once daily in the CICU to assess for ischemic or rhythmic disorders. Transthoracic echocardiography was performed for all patients following coronary intervention and prior to discharge from the hospital.

### 2.3. Coronary Angiography Evaluation

All patients received antithrombotic therapy prior PCI, including 180 mg of Ticagrelor© or 300 to 600 mg of Clopidogrel©, always combined with 250 mg of Aspirin© and unfractionated or low weight molecular heparin (dosage was determined according to weight and kidney function). Loading dose of P2Y12 inhibitors was administrated after coronary anatomy evaluation for NSTEMI and as soon as possible in STEMI according to guidelines [3,4]. After coronary angiography, the indication for immediate PCI or medical treatment was evaluated by the interventional cardiologist according to guidelines and local protocols [3,4]. Referral for coronary artery bypass graft surgery was typically decided by our team including cardiovascular surgeons and cardiologists. PCI was commonly performed ad hoc for the culprit lesion in NSTEMI. Non-culprit lesions were generally treated in a deferred manner.

### 2.4. Study Endpoints

The primary endpoint, evaluated in high- and low-risk groups, was the incidence of at least one in-hospital major adverse event. Major outcomes were defined as all-cause mortality, serious neurological or bleeding complications (>BARC 2), hemodynamic instability requiring medical intervention, heart failure requiring medical intervention, sustained, or poorly tolerated ventricular rhythm or conduction disorders requiring therapeutic intervention, chest pain recurrence requiring new coronary angiography or revascularization, and any secondary transfer to CICU for any reason.

Secondary endpoints included one-month all-cause mortality, rehospitalization and total hospitalization length of stay. All patients were given full study information and written consent was obtained. The protocol was approved by the local ethics committee and the institutional regulatory authorities (*Institutional Review board approval number: IRB-Montpellier_2020_05_202000472*) and was undertaken following the international epidemiological and clinical trials guidelines. The study was conducted according to the principals of the Declaration of Helsinki and was registered with ClinicalTrials.gov (ID 04378504) on 4 May 2020.

### 2.5. Statistical Analysis

Data were collected using clinical, biological, ECG and echocardiography evaluation according to the usual practice in our center. No additional testing or biological samples were specifically required for the study.

Assuming an incidence of 3% serious non-fatal events in the low-risk group and 15% of serious events in the high-risk group, inclusion of at least 210 patients was required (for a power of 90% and an alpha risk of 5%). Patient’s characteristics are presented using mean and standard deviation (SD) for continuous variables and frequencies and proportions for categorical variables. The chi-square test or Fisher’s exact test was used to compare categorical variables between groups (high- and low-risk). The Student *t*-test or the Wilcoxon–Mann–Whitney (WMW) test was used to compare continuous variables. The negative and positive predictive values of adverse events in each group were also calculated. All analyses were conducted using SAS V9.1 (The SAS System, Cary, NC, USA) and the statistical significance threshold was set at 5%.

## 3. Results

### 3.1. Study Population

**Figure 1:**
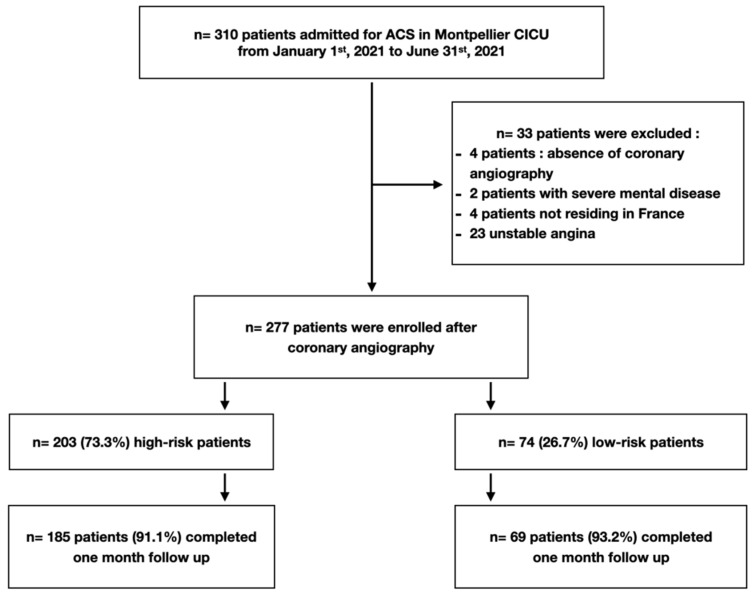
Study flow chart.

Among 308 patients admitted for ACS within the study period, 277 were studied including 174 STEMI patients (62.8%) and 103 NSTEMI patients (37.2%) (Figure 1). Population baseline characteristics are presented in Table 2. At admission, median left ventricular ejection fraction was 50% (45–60) and 17 patients (6.1%) had unstable hemodynamics.

**Figure 2:**
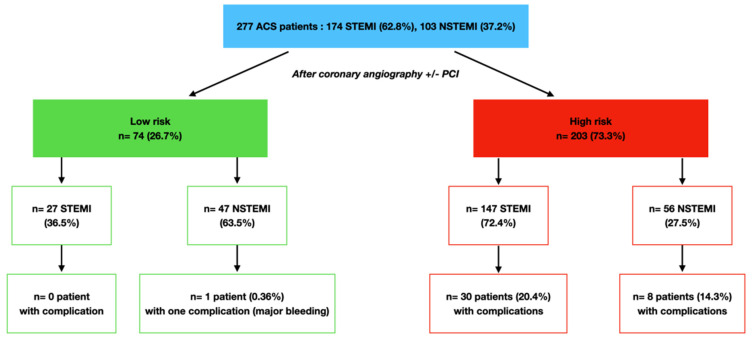
Major in-hospital outcomes according to risk stratification.

After coronary angiography evaluation and PCI when required, 74 (26.7%) patients were classified in the low-risk group and 203 (73.3%) patients in the high-risk group according to our predefined criteria (Table 1 and Table 2, Figure 1 and Figure 2). Among STEMI patients, 27 (15.5%) were classified as low-risk and 147 (85.5%) patients were classified as high-risk. Among NSTEMI patients, 47 (45.6%) were classified as low-risk and 56 (54.4%) as high-risk. PCI was required for 260 patients (93.9%), 15 patients (5.4%) required surgery and 2 patients were managed medically (0.7%). High-risk patients had lower left ventricular ejection fraction (LVEF) (*p* < 0.01), higher rates of multivessel disease (*p* < 0.01) and left main disease (*p* = 0.05); Table 2.

### 3.2. End Points

The primary endpoint was reached in 39 patients (14.1%). While 38 patients (18.7%) from the high-risk group reached the primary endpoint, only 1 patient (1.3%) in the low-risk group had one major outcome (no fatal major bleeding event); *p* < 0.01 (Figure 2). Only patients included in the high-risk group had unstable hemodynamics or rhythmic state, high degree conduction disorders or sustained ventricular tachycardia; Table 3. Number of major complications per patient and according to risk stratification are presented in Table 4.

Among the high-risk STEMI patients (n = 147), 20 patients (13.6%) reached the primary endpoint, while 8 patients among high-risk NSTEMI patients (n = 56) (14.3%) reached this endpoint; *p* = 0.9.

Among the low-risk patients, 98.6% had no complications (NPV = 98.6%) (IC95% [96.0–100%]), and no life-threatening events was observed. No patient in the low-risk STEMI subgroup had adverse events (NPV 100%) (IC95% [100–100%]). One patient (1.4%) in the low-risk NSTEMI subgroup had a major outcome with major digestive bleeding requiring transfusion (2 units of red blood cells) and was switched from ticagrelor to clopidogrel (NPV 97.9% (IC95% [93.2–100%])).

Hospitalization length of stay was significantly shorter in the low-risk vs. high risk group (3.0 (2.0–4.0) vs. 5.0 (4.0–6.0); *p* < 0.01). One-month all-cause mortality rate was similar between the low-risk group (n = 0 (0.0%)) and the high-risk group (n = 6 (2.3%)); *p* = 0.4, as well as the one-month rehospitalization rate (n = 4 (5.8%) and n = 14 (7.6%), respectively); *p* = 0.8.

## 4. Discussion

We prospectively evaluated in-hospital outcomes in ACS patients (NSTEMI and STEMI) benefitting from contemporary care management and obtained two main findings:

1. Approximately 1/4 of all ACS and 1/2 of NSTEMI patients may be considered at low risk of in-hospital major outcomes based on simple and routinely assessed clinical and angiographic criteria;
2. Following early invasive strategy and PCI for the vast majority of patients, a low rate of complications with no life-threatening event, rhythmic or conduction disorders was observed in the low-risk group (NPV 98.6% for all ACS and NPV 100% in the STEMI group).

### 4.1. Low vs. High-Risk ACS Patients

In the present study, about 25% of our total population was classified as having a low risk of events according to predefined criteria derived from the European guidelines for ACS management [3,4]. Compared to high-risk patients, low-risk patients were younger, and mainly presented with NSTEMI (around 2/3). As expected, coronary artery disease was less severe, LVEF was higher in the low-risk group and PCI was performed in most of the cases (98.7%). Several mortality risk scores have been developed for ACS patients and were mostly validated by observational data [9,10,11,12]. For patients with unstable angina or NSTEMI, these scores were validated for prognostic stratification and therapeutic decision-making but did not use coronary anatomy analysis in their algorithm [9,10]. The Global Registry of Acute Coronary Events (GRACE) risk score has been used in patients with ACS to assess either short or long-term outcomes [12,13]. However, performance of this risk score was controversial and the new NSTEMI guidelines have retrograded its use from I to IIA for prognostic stratification [4]. Furthermore, this risk score has not been evaluated for predicting in-hospital severe events, indications for ECG monitoring or CICU admission in patients presenting with ACS. In the ACTION registry evaluating 29,973 NSTEMI patients, 9 clinical variables (heart failure, heart rate, systolic blood pressure, troponin, serum creatinine, prior revascularization, chronic lung disease, ST-segment depression and age) were identified as predictors of complication requiring CICU management [14]. However, this registry was conducted prior our modern era including early angiography and PCI according to the 2020 NSTEMI guidelines [4]. In a retrospective analysis, the ACTION score was described as an effective tool to identify the need for CICU care for patients with NSTEMI and early invasive strategy according to the 2020 guidelines [15,16]. The CCC-ACS CS score, which was developed from a largescale dataset of unselected ACS Chinese patients, quantifies the risk of in-hospital death for patients with ACS at early medical contact and may facilitate clinical decision-making but did not select patients with or without need of CICU admission [17]. Thus, current value of risk scores following early PCI which broadly contributes to stabilize the patient, may be reevaluated in a prospective study. In the present study, troponin level or ECG changes were not included for risk stratification following PCI, as recommended in the 2020 guidelines to predict risk of events [4].

### 4.2. Early Invasive Strategy for Patient Stratification

Contemporary practice for NSTEMI management has changed since 2020 and includes an early invasive strategy (<24 h) in stable patients and concomitant use of evidence-based medications, such as potent antithrombotic and antiplatelet therapy, statins and B-blockers [3,4,8]. Among ACS patients managed with an early invasive strategy, the ACUITY study emphasizes the prognostic importance of the diagnostic angiogram in patient risk stratification, providing important added independent predictive value for 30-day and 1-year ischemic outcomes [18]. In our study, low rate of events with no life-threatening events was observed in the low-risk group with risk stratification including diagnostic angiogram and PCI result. Meta-analyses have consistently reported that an early invasive strategy is associated with a lower risk of recurrent/refractory ischemia and a shorter length of hospital stay [19,20,21,22]. In the meta-analysis of Jobs et al., early evaluation of coronary anatomy and PCI when required, was found to reduce mortality only in higher risk patients [22]. In the present study, both early coronary angiography and high rate of PCI may probably have contributed to the low rate of events of our NSTEMI population. In our STEMI population, 15% of the patients were considered at low risk using strict selection criteria including early or spontaneous, successful reperfusion with no adverse events at follow-up in this group and particularly no rhythmic events. Early spontaneous reperfusion was previously associated with favorable short- and long-term prognosis following STEMI [23]. Conversely, incidence of in-hospital adverse events in the high-risk group was high (18.7% in the total population and 20.4% in the STEMI group) and mainly related to rhythmic disorders or hemodynamics instability and required CICU admission or at least ECG monitoring. These patients may be characterized by simple clinical characteristics (age and LVEF) and angiographic patterns (multivessel disease, left main disease and lower rate of PCI).

The 2017 European guidelines recommend at least 24 h monitoring in CICU for patients with STEMI [3]. For NSTEMI, rhythm monitoring up to 24 h or to PCI is a strong recommendation (level 1) but with a low level of evidence (level C), related more to expert consensus than to evidence-based data [4]. The Premier database study evaluating 114,136 hospitalizations for ACS in 307 hospitals over 2 years, revealed a marked variation in CICU admission across centers [5]. Another observational study showed that ultimately 61% of ACS admissions received no advanced CICU therapies [24]. The results of the ACTION registry suggested that CICU triage was largely driven by local practices and practitioner preference [25]. A transfer back, the same day, to a referring non-PCI hospital of patients with ACS and successful primary PCI without ongoing ischemia, severe heart failure or arrhythmias, not requiring vasoactive drugs, mechanical support or further early revascularization is allowed in guidelines [3]. Our results suggest that selected NSTEMI (half of patients) and STEMI patients, may be identified as at very low risk of adverse events and may probably not require systematic CICU admission or ECG monitoring following early angiography evaluation and successful PCI when required. As anticipated, total hospitalization length of stay was significantly shorter in the low-risk group and may impact hospitalization costs.

Finally, this study needs to be considered in light of some limitations. The first limitation is the single center design limiting the extension of our results to other centers. Secondly, our study was observational, and all patients were admitted in CICU. Thus, our findings need to be confirmed in a randomized study without CICU admission for selected low-risk patients. Lastly, proportion of STEMI was high in our population, possibly related to inclusion during the COVID epidemic period. Previous studies have shown a decrease in CICU admissions during COVID period, for all types of acute coronary syndrome, including both STEMI and NSTEMI, but even more frequently for NSTEMI [26,27].

In conclusions, identification of ACS with very low risk of adverse events is feasible considering simple clinical parameters after coronary angiography evaluation, optimal antithrombotic therapy and successful PCI in most of cases. Incidence of adverse outcomes was low with no fatal complications and no severe rhythmic event observed in “low-risk” ACS (NSTEMI and STEMI). These selected patients (25% of the total ACS population and nearly half of NSTEMI) may probably not require systematic CICU admission.

The appropriate use of the resource intensive setting is a major issue in clinical practice, and CICU triage optimization is necessary. Early coronary anatomy evaluation and PCI, when required, associated with simple clinical parameters may select high vs. low risk patients regarding risk of in-hospital severe events and need of CICU admission. These results, however, need to be confirmed in a randomized and multicenter study. The cost-effectiveness of this strategy, particularly regarding hospitalization length, must also be evaluated.

## Figures and Tables

**Table 1:** Predefined criteria for high versus low-risk groups of NSTEMI and STEMI patients.

	Low-Risk Group	High-Risk Group
NSTEMI and STEMI patients		
Age > 80 years	-	+
Unstable hemodynamics ^@^	-	+
Severe comorbidities ^▲^	-	+
Left ventricular ejection fraction < 40%	-	+
Rhythmic state requiring specific therapeutic intervention ^&^	-	+
Failure of reperfusion or unsatisfactory result of PCI ^⌀^	-	+
Severe residual coronary lesions requiring further revascularization ^⌀^	-	+
High bleeding risk *	-	+
STEMI patients		
Early and successfully reperfused STEMI ^#^	+	-

^@^ Mean blood pressure < 65 mmHg, need for hemodynamic support or fluid infusion. ^▲^ Severe renal impairment (creatinine clearance < 20 mL/min), chronic lung disease with O_2_ dependence, any comorbidity requiring specific care. ^&^ specific medications or urgent cardioversion. ^⌀^ Residual stenosis >30%, TIMI flow < grade 3, residual dissection, or non-occlusive thrombus on final angiography. * CRUSADE Score > 41. ^#^ Less than 3 h after the onset of pain, TIMI 3 flow and loading dose of aspirin and P2Y12 before PCI.

**Table 2:** Study population baseline characteristics.

	Total Population n = 277	Low-Risk Group n = 74	High-Risk Group n = 203	*p*-Value
Age (years), median	66 (56–75)	63 (54–70)	67 (57–78)	<0.01
Male sex, n (%)	208 (75.1)	61 (82.4)	147 (72.4)	0.09
Body mass index (kg/m^2^), median	26 (24–29)	26 (24–29)	26 (23–29)	0.27
Hypertension, n (%)	122 (44.0)	27 (36.5)	95 (46.8)	0.13
Active smoker, n (%)	115 (41.5)	35 (47.3)	80 (39.4)	0.24
Prior MI, n (%)	45 (16.3)	6 (8.1)	39 (19.2)	0.03
Severe pulmonary disease, n (%)	12 (4.3)	2 (2.7)	10 (5)	0.52
Severe chronic renal disease ^▲^, n (%)	6 (2.2)	0 (0)	6 (2.9)	0.49
Severe chronic lung disease, n (%)	0 (0)	0 (0)	0 (0)	1
Oral anticoagulants, n (%)	16 (5.8)	0 (0)	16 (7.9)	0.01
Type of AMI				
STEMI, n (%)	174 (62.8)	27 (15.5)	147 (84.5)	<0.01
NSTEMI, n (%)	103 (37.1)	47(45.6)	56 (54.4)	<0.01
LVEF, median	50 (45–60)	60 (50–60)	50 (40–55)	<0.01
Antiplatelet therapy				
Clopidogrel, n (%)	22 (7.94)	4 (5.4)	18 (8.9)	0.3
Ticagrelor, n (%)	255 (92.1)	70 (94.6)	185 (91.1)	0.3
Coronary angiogram				
Single vessel disease, n (%)	95 (34.3)	36 (48.7)	59 (29.1)	<0.01
Multivessel disease, n (%)	182 (65.7)	38 (51.4)	144 (70.9)	<0.01
LM disease	10 (3.6)	0 (0.0)	10 (4.9)	0.05
LAD disease	143 (51.6)	40 (54.1)	103 (50.7)	0.6
Circumflex disease	57 (20.6)	17 (22.9)	40 (19.7)	0.6
RCA disease	99 (35.7)	21 (28.4)	78 (38.4)	0.1
Stent thrombosis, n (%)	4 (1.4)	0 (0.0)	4 (2.0)	0.2
Revascularization strategy				
PCI, n (%)	260 (93.9)	73 (98.7)	187 (92.1)	0.05
Bypass surgery, n (%)	15 (5.4)	0 (0.0)	15 (7.4)	0.02
Medical therapy only	2 (0.7)	1 (1.3)	1 (0.5)	0.5

Values are n (%) or median (interquartile range). LAD: left anterior descending; LM: left main; MI: myocardial infarction; PCI: percutaneous coronary intervention; RCA: right coronary artery. ^▲^ Cl < 30 mL/min.

**Table 3:** In-hospital major outcomes (primary endpoint).

	Total Population n = 277	Low-Risk Group n = 74	High-Risk Group n = 203	*p*-Value
Total in-hospital complications, n (%) *	38 (14.1)	1 (1.3)	37 (18.2)	<0.01
**Unstable hemodynamic state, n (%)**	17 (6.1)	0 (0.0)	17 (8.4)	<0.01
**Pericardial effusion requiring treatment, n (%)**	7 (2.5)	0 (0.0)	7 (3.5)	0.11
**Sustained or poorly tolerated ventricular arrhythmia, n (%)**	13 (4.7)	0 (0.0)	13 (6.4)	0.03
**Severe conduction disorders, n (%)**	11 (4.0)	0 (0.0)	11 (5.2)	0.04
**Chest pain recurrence requiring coronary angiography, n (%)**	7 (2.5)	0 (0.0)	7 (3.5)	0.1
**Death from any cause, n (%)**	6 (2.2)	0 (0.0)	6 (3.0)	0.4
**Heart failure, n (%)**	7 (2.5)	0 (0.0)	7 (3.5)	0.1
**Major bleeding, n (%)**	3 (1.1)	1 (1.3)	2 (1.0)	1
**Stroke, n (%)**	1 (0.4)	0 (0.0)	1 (0.5)	1
**Secondary transfer to CICU, n (%)**	3 (1.1)	0 (0.0)	3 (1.5)	0.6

* Number of patients who presented at least one major complication.

**Table 4:** Number of major events per patient in low- and high-risk groups.

	Number of Events							
	0	1	2	3	4	5	6	Total
Low-risk, n (%)	73 (98.6)	1 (1.4)	0 (0.0)	0 (0.0)	0 (0.0)	0 (0.0)	0 (0.0)	74 (26.8)
High-risk, n (%)	165 (81.3)	19 (9.4)	10 (4.9)	5 (2.5)	2 (1.0)	1 (0.5)	1 (0.5)	203 (73.2)
Total, n (%)	238 (85.9)	20 (7.2)	10 (3.6)	5 (1.8)	2 (0.7)	1 (0.4)	1 (0.4)	277
